# Mapping BCG vaccination coverage in Ethiopia between 2000 and 2019

**DOI:** 10.1186/s12879-022-07547-4

**Published:** 2022-06-23

**Authors:** Kendalem Asmare Atalell, Mulat Asrade Alemayehu, Nahom Worku Teshager, Getaneh Mulualem Belay, Tewodros Getaneh Alemu, Degefaye Zelalem Anlay, Amare Wondim, Kefyalew Addis Alene

**Affiliations:** 1 Department of Pediatrics and Child Health Nursing, School of Nursing, College of Medicine and Health Sciences, University of Gondar, Gondar, Ethiopia; 2 Department of Pediatrics and Child Health, School of Medicine, College of Medicine and Health Sciences, University of Gondar, Gondar, Ethiopia; 3 Senior Consultant Pediatrician at the University of Gondar Comprehensive Specialized Hospital, Gondar, Ethiopia; 4 Unit of Community Health Nursing, School of Nursing, College of Medicine and Health Sciences, University of Gondar, Gondar, Ethiopia; 5 Telethon Kids Institute, Nedlands, WA Australia; 6 Faculty of Health Sciences, Curtin University, Bentley, WA Australia

**Keywords:** BCG, Coverage, Ethiopia, Immunization, Spatiotemporal

## Abstract

**Introduction:**

The Bacille-Calmette–Guerin (BCG) vaccination remains the primary strategy to prevent severe disseminated TB in young children, particularly in high TB-burden countries such as Ethiopia. Accurate knowledge of vaccination coverage in small geographical areas is critically important to developing targeted immunization campaigns. Thus, this study aimed to investigate the spatiotemporal distributions and ecological level determinants of BCG vaccination coverage in Ethiopia.

**Method:**

Bacille-Calmette–Guerin immunization coverage and geographical information data were obtained from five different Demographic and Health Surveys, conducted in Ethiopia between 2000 and 2019. Data for independent variables were obtained from publicly available sources. Bayesian geostatistical models were used to predict the spatial distribution of BCG vaccination coverage in Ethiopia.

**Result:**

The overall national BCG vaccination coverage between 2000 and 2019 was 65.5%. The BCG vaccine coverage was 53.5% in 2000, 56.9% in 2005, 64.4% in 2011, 79.6% in 2016, and 79.0% in 2019. BCG vaccination coverage increased by 47.6% in Ethiopia from 2000 to 2019, but substantial geographical inequalities in BCG coverage remained at sub-national and local levels. High vaccination coverage was observed in northern, western, and central parts of Ethiopia. Climatic and demographic factors such as temperature, altitude, and population density were positively associated with BCG vaccination coverage. Whereas, healthcare access factors such as distance to health facilities and travel time to the nearest cities were negatively associated with BCG vaccine coverage in Ethiopia.

**Conclusion:**

Despite substantial progress in national BCG vaccination coverage, marked spatial variation in BCG coverage persists throughout the country at sub-national and local levels. Healthcare access and climatic and demographic factors determined the spatial distribution of BCG vaccination coverage. Maintaining a high level of vaccination coverage across geographical areas is important to prevent TB in Ethiopia.

**Supplementary Information:**

The online version contains supplementary material available at 10.1186/s12879-022-07547-4.

## Introduction

The Bacille-Calmette–Guerin (BCG) vaccine is the only vaccine currently available for the prevention of tuberculosis (TB), a bacterial disease that kills more than one million people globally every year [1, 2]. The BCG vaccine contains a live attenuated *Mycobacterium bovis* that can prevent severe forms of TB such as meningitis and disseminated TB [3, 4]. BCG vaccination is universally provided for all newborn babies in high TB-burden countries such as India and Ethiopia. In low TB incidence countries, it is targeted to specific TB risk groups [5, 6].

Although various efforts have been made to improve access and coverage of BCG vaccination, TB remains a major health problem among children in developing countries including Ethiopia [7, 8]. The World Health Assembly endorsed the Global Vaccine Action Plan 2011–2020 as a framework for maximizing the advantages of vaccination and realizing a vision of universal access to vaccines and immunization with 90% coverage [9]. As part of this global plan, Ethiopia has made several efforts to improve the national BCG vaccination coverage. In the last few decades, BCG vaccination coverage has been enhanced through the combined efforts of the Reaching Every District approach, a health extension program, and the introduction of Enhanced Routine Immunization Activities in Ethiopia [10]. However, despite all these tremendous efforts, the BCG vaccination coverage remains low in the country [11].

The coverage of BCG vaccination can be influenced by various factors including the socio-demographic and economic status of the mother [12–14]. Previous studies conducted in developed countries showed that the rate of BCG vaccination was associated with family-related characteristics such as wealth index, literacy rate, and healthcare access [15, 16]. Few primary studies have been also conducted in Ethiopia to examine the determinants of BCG vaccination coverage. However, all the studies did not consider geographic variation in the estimation of BCG vaccination uptake [12, 13, 17]. Previous studies were not also considered ecological level factors associated with BCG vaccination at the subnational and local levels. However, accurate knowledge of vaccination coverage in small geographical areas is critically important for designing targeted immunization campaigns. Identifying areas with low vaccination coverage is important for immunization programs, particularly in Ethiopia and other low-income countries for the rational allocation of resources. The information is also important to measure the efforts and achievements of vaccination programs at the local level. Thus, this study aimed to investigate the spatial pattern and drivers of BCG vaccination among children in Ethiopia,

## Methods

### Study setting

The study was conducted in Ethiopia using an ecological study design. Ethiopia is the second-most populous country in Africa, with an estimated population size of more than 115 million people in 2020 [18]. There are marked differences in population structure, socioeconomic conditions, disease burden, and climatic conditions across the country. Ethiopia has a surface area of approximately 1.1 million square kilometers and a population density of more than 215 people per square kilometer [18]. It has a variety of geographical features altitudes ranging from 125 m below sea level in Afar depression to 4533 m above sea level in Ras Dejen, Amhara region. Ethiopia has a three-tier health system through which immunizations and other health care services are delivered. More than 84% of the population resides in rural parts of the country with low healthcare access. Universal BCG vaccination at birth is part of the national health program in Ethiopia.

### Data source

Data for BCG immunization coverage was obtained from the Ethiopian Demographic and Health Survey (EDHS). The EDHS contains five different surveys which have been conducted every 5 years between 2000 and 2019. The survey was conducted using nationally representative samples that provided estimates for urban and rural areas. Global Positioning System (GPS) coordinates for each survey location were also available in the EDHS.

Geospatial covariate data were obtained from several publicly available sources with a resolution of 1 km^2^. Climatic variables such as mean monthly temperature and mean monthly precipitation were accessed from the WorldClim website [19] and altitude data was obtained from the Shuttle Radar Topography Mission (SRTM) [20]. Travel time to cities in minutes and access to health care facilities were retrieved from the Malaria Atlas Project (MAP) [21]. Population density and distance to waterbody data were accessed from WorldPop [22] and Global Lakes and Wetlands Database (GLWD), respectively. The covariates were selected based on potential associations with BCG vaccination coverage, as obtained from previous literature. The choice of these variables was also based on the availability of high-resolution country-wide data. The polygon shapefile for the Ethiopian administrative boundaries was obtained from the Global Administrative Areas (GADEM) free online database.

### Data processing and analysis

The dependent variable for this study was BCG vaccination coverage, which was operationally defined as the number of children who received the BCG vaccine divided by the total number of children born within five years preceding the survey. Descriptive statistics were first carried out to summarize BCG immunization coverage at the regional level.

### Spatial analysis

The Bayesian model-based geostatistics was used to generate a spatially continuous estimate of the national BCG immunization coverage mapped at a resolution of 1 km^2^. The binomial regression model was fitted within the Bayesian framework to the BCG immunization coverage of both fixed effects and geostatistical random effects. Five models were constructed separately for each year of BCG immunization coverage data (i.e., 2000, 2005, 2011, 2016, and 2019). The model for immunization coverage was the same for all five data sets. A spatial binomial regression model was fitted for immunization coverage using geostatistical random effects and covariate fixed effects for mean annual temperature, mean annual precipitation, altitude, travel time to the nearest city, distance to the nearest health facilities, distance to the water body, and population density [23]. The BCG immunization coverage was taken at each surveyed location *j* as the outcome variable, which was assumed to follow a binomial distribution;

$${Y}_{j}\sim Binomial ({n}_{j},{p}_{j})$$

where ${Y}_{j}$ is the observed immunization coverage, ${n}_{j}$ is the total number of vaccinated and unvaccinated children and ${p}_{j}$ is the predicted BCG immunization coverage at location$j$. Mean predicted BCG immunization coverage was modeled via a logit link function to a linear predictor defined as: .

$$\log it \left( {p_{j} } \right) = \alpha + \sum\limits_{z = 1}^{z} {\beta_{z} X_{z,j } + \zeta_{j } }$$

where *α* is the intercept, *β* is a matrix of covariate coefficients, ${\varvec{X}}$ is a design matrix of $z$ covariates, and ${\zeta }_{j}$ are spatial random effects modeled using a zero-mean Gaussian Markov random field (GMRF) with a Matérn covariance function. The covariance function was defined by two parameters: the range $\rho$, which represents the distance beyond which correlation becomes negligible (about 0.1 km), and $\sigma$, which is the marginal standard deviation [24, 25]. Non-informative priors were used for *α* (uniform prior with bounds – ∞ and ∞), and we set normal priors with mean = 0 and precision (the inverse of the variance) = 1 × 10^–4^ for each *β*. We used default priors for the parameters of the spatial random field [26]. Parameter estimation was done using the Integrated Nested Laplace Approximation (INLA) approach in R (R-INLA) [24, 25]. Sufficient values (i.e., 150,000 samples) from each simulation run for the variables of interest were stored to ensure full characterization of the posterior distributions.

Predictions of BCG immunization coverage at unsampled locations were made at 1 km^2^ resolution by interpolating the spatial random effects and adding them to the sum of the products of the coefficients for the spatially variant fixed effects at each prediction location [27]. The intercept was added, and the overall sum was back-transformed from the logit scale to the prevalence scale, providing prediction surfaces that show the estimated immunization coverage for all prediction locations.

The Watanabe Applicable Information Criterion (WAIC) statistic was used to select the best-fitting model.

## Result

### National and regional coverage of BCG vaccine in Ethiopia

The national and regional coverage of the BCG vaccine in Ethiopia between 2000 and 2019 is described in (Table 1). The average national BCG vaccine coverage for all five EDHS data was 65.5%. The coverage for each EDHS was 53.5% in 2000, 56.9% in 2005, 64.4% in 2011, 79.6% in 2016, and 79.0% in 2019. While high regional coverage of the BCG vaccine was observed in Addis Ababa, Tigray, and Dire Dawa, low coverage was observed in Somali and Afar regions (Table 1).

**Table 1:** The national and regional BCG vaccine coverage in Ethiopia between 2000 and 2019

Region	Coverage % (2000)	Coverage % (2005)	Coverage % (2011)	Coverage % (2016)	Coverage % (2019)	Coverage % (2000–2019)
Tigray	77.6	77.5	91.4	89.5	92.5	85.1
Afar	26.3	32.3	37.8	60.6	62.0	45.1
Amhara	50.8	64.2	68.9	83.4	85.4	66.6
Oromia	49.2	51.4	57.2	73.7	77.1	58.7
Somali	36.7	23.8	42.5	70.8	63.0	51.2
Benishangul	41.2	48.3	68.5	86.3	82.6	65.3
SNNPR	44.2	58.7	64.4	84.6	77.6	63.3
Gambela	44.0	50.7	54.3	78.2	78.8	60.7
Harari	78.9	64.7	74.8	81.4	81.1	76.2
Addis Ababa	93.7	87.6	95.0	97.0	97.6	94.1
Dire Dawa	74.1	72.3	82.6	94.0	93.8	83.3
Ethiopia	**53.5**	**56.9**	**64.4**	**79.6**	**79.0**	**65.5**

*SNNPR* Southern, Nations, Nationalities and Peoples Region

### Trends of BCG vaccine coverage

**Fig. 1:**
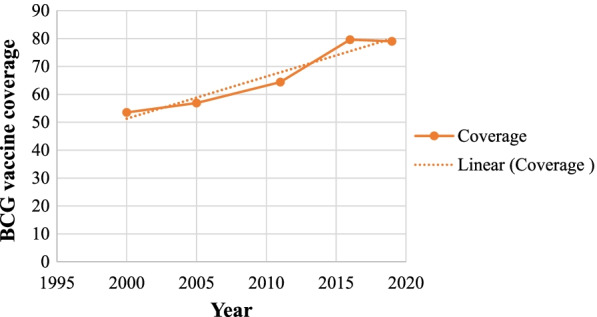
Trend analysis graph of BCG immunization coverage in Ethiopia between 2000 and 2019.

The temporal trends of the BCG vaccine coverage in Ethiopia from 2000 to 2019 are described in Fig. 1. In the first four surveys, the coverage was increased from 53.5% in 2000 to 79.6% in 2016, with a faster increment between 2011 and 2016. However, BCG vaccine coverage slightly declined from 79.6% in 2016 to 79% in 2019 (Fig. 1).

### Spatial clustering of BCG vaccine coverage

**Fig. 2:**
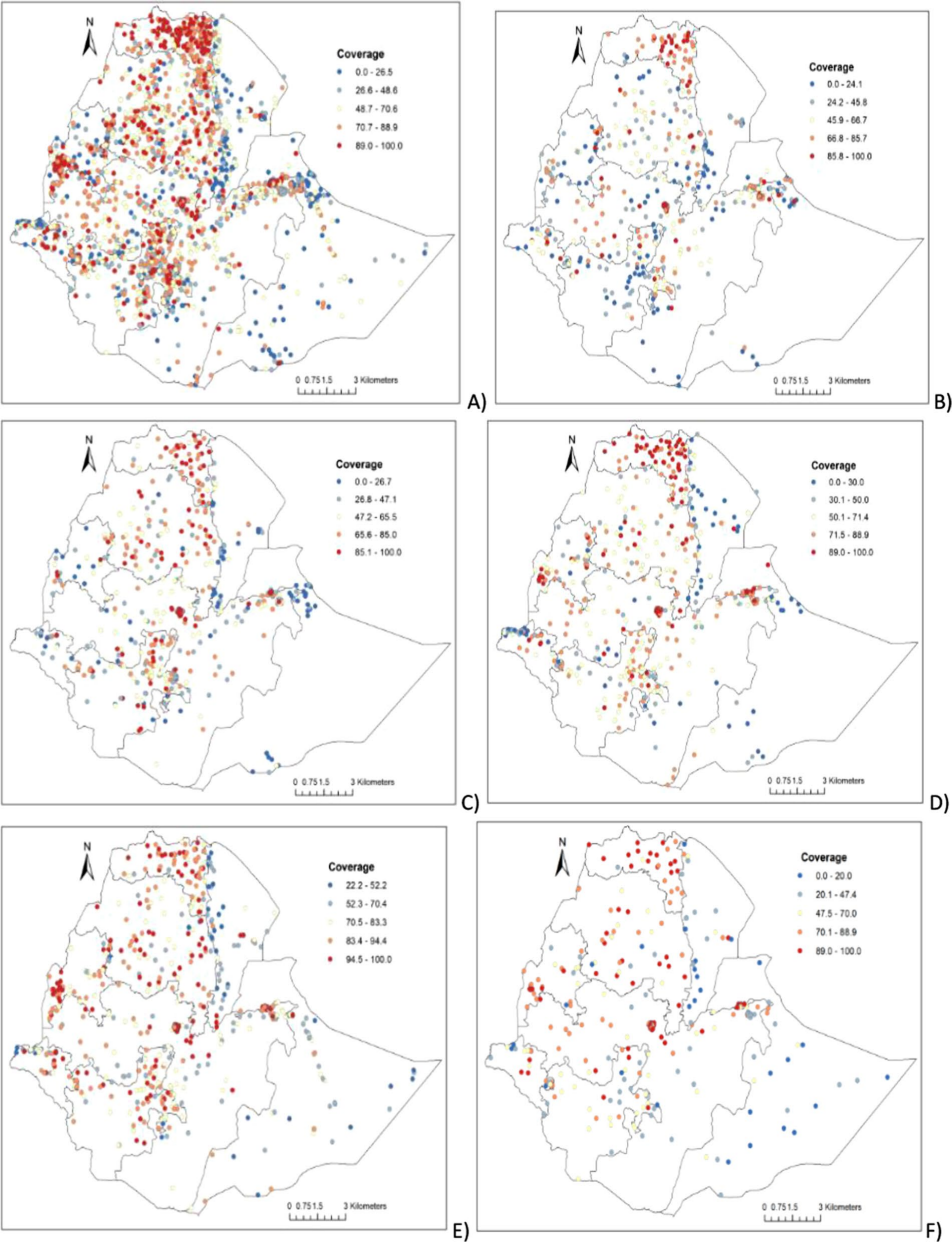
Geospatial points and BCG vaccine coverage in Ethiopia: **A** 2000–2019, **B** 2000, **C** 2005, **D** 2011, **E** 2016 and **F** 2019.

Significant spatial variations of BCG vaccine coverage were observed at sub-national and local levels in Ethiopia. High BCG vaccine coverage was observed in Northern (Tigray region), Central (Addis Ababa), and Eastern (Dire Dawa), whereas lower BCG vaccine coverage was observed in Eastern (Afar and Somali), Western (Gambela and Benshangul_gumuz), and Southern (Oromia and SNNPR) parts of Ethiopia (Fig. 2).

**Fig. 3:**
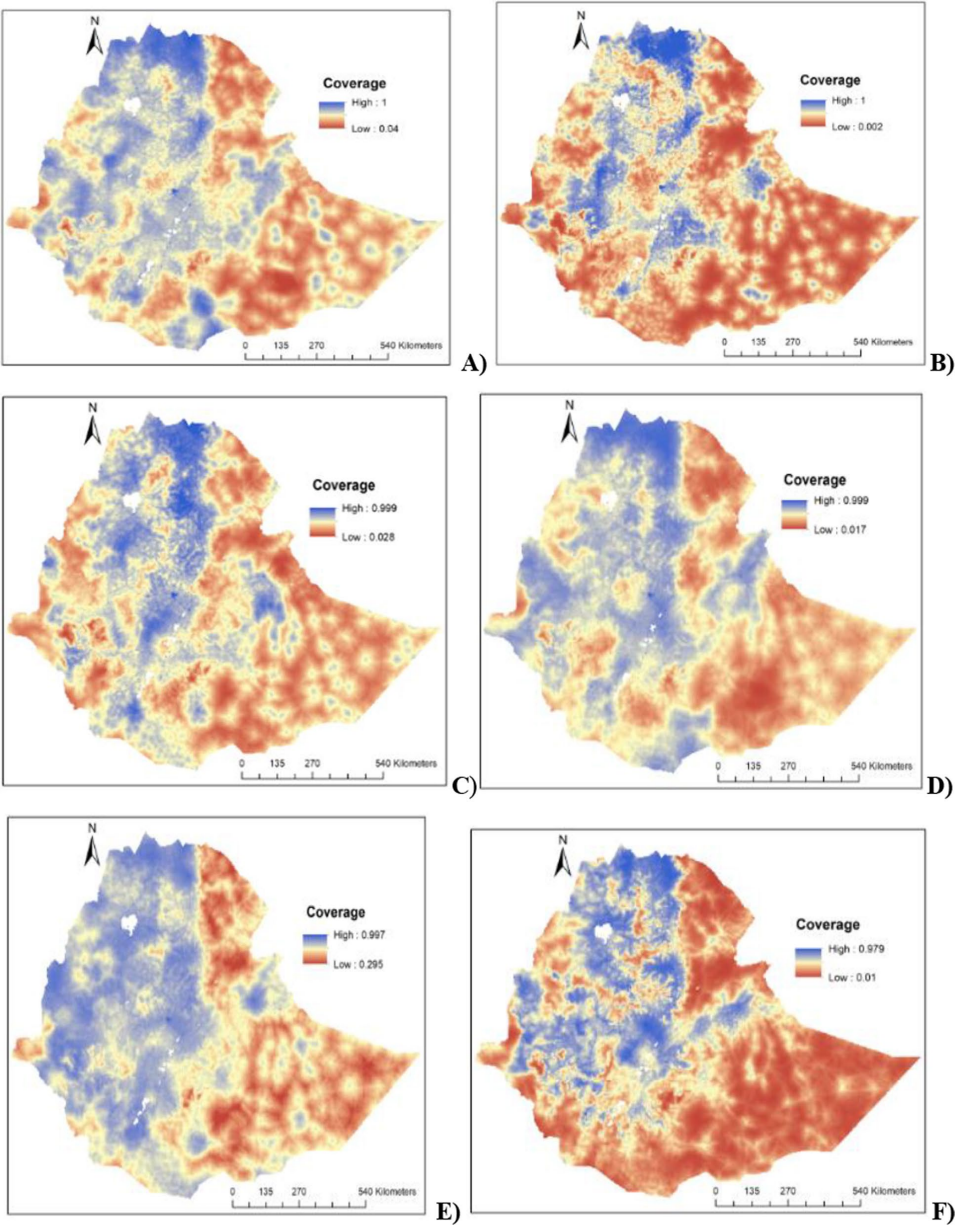
The predicted geospatial map for BCG immunization coverage in Ethiopia: **A** 2000–2019, **B** 2000, **C** 2005, **D** 2011, **E** 2016 and **F** 2019.

The predicted BCG vaccine coverage for the overall and each individual EDHSs were delineated in Fig. 3. Highest predicted BCG vaccine coverage was observed in Northern, Northwestern, and central parts of Ethiopia, and lower BCG vaccine coverage was observed in Southern, Eastern, and Northeastern parts of the country (Fig. 3).

### Drivers of BCG immunization coverage

The Bayesian geostatistical model was used to investigate the association between BCG vaccine coverage and covariates. Temperature was positively associated with BCG vaccine coverage in 2000 [mean regression coefficient (β): 0.63; 95% credible interval (95% CrI): 0.11, 1.15] and 2019 (β: 2.23; 95% CrI: 1.20, 3.27). Altitude was also positively associated with BCG vaccine coverage in 2019 (β: 2.61; 95% CrI: 1.57, 3.66). Furthermore, the population density was positively associated with BCG vaccine coverage in all EDHSs. Whereas travel time to the nearest cities in minutes and distance to health facilities were negatively associated with BCG vaccine coverage in all EDHS (Table 2).

**Table 2:** Regression coefficient mean and 95% credible intervals (CrI) of covariates included in a Bayesian spatial model with Binomial response for the BCG immunization coverage in Ethiopia between 2000 and 2019

Covariates	Full immunization coverage Regression coefficients of Mean (95% CrI)					
	**2000**	**2005**	**2011**	**2016**	**2019**	**2000–2019**
Temperature	**0.63 (0.11, 1.15)**	0.36 (− 0.14, 0.86)	0.18 (− 0.37, 0.73)	0.27 (− 0.30, 0.82)	**2.23 (1.20, 3.27)**	0.17 (− 0.08, 0.41)
Precipitation	0.10 (− 0.12, 0.33)	− 0.19 (− 0.45, 0.06)	0.12 (− 0.14, 0.39)	0.16 (− 0.07, 0.36)	0.29 (− 0.02, 0.59)	− 0.05 (− 0.18, 0.08)
Altitude	0.49 (− 0.05, 1.03)	0.44 (− 0.07, 0.95)	0.29 (− 0.28, 0.86)	0.35 (− 0.23, 0.91)	**2.61 (1.57, 3.66)**	0.08 (− 0.18, 0.33)
Travel time to the nearest city	**− 0.27 (− 0.40, − 0.15)**	**− 0.24 (− 0.38, − 0.10)**	**− 0.21 (− 0.32, − 0.10)**	− 0.13 (− 0.27, 0.01)	**− 0.55 (− 0.84, − 0.26)**	**− 0.12 (− 0.18, − 0.07)**
Population density	**0.06 (0.05, 0.07)**	**0.05 (0.03, 0.06)**	**0.04 (0.03, 0.06)**	**0.03 (0.02, 0.04)**	**0.01 (0.004, 0.03)**	**0.05 (0.05, 0.06)**
Distance to water body	− 0.04 (− 0.10, 0.02)	0.01 (− 0.05, 0.06)	− 0.05 (− 0.11, 0.004)	− 0.05 (− 0.11, 0.02)	− 0.08 (− 0.21, 0.05)	− 0.002 (− 0.03, 0.02)
Distance to health facilities	**− 0.82 (− 1.06, − 0.58)**	**− 0.47 (− 0.68, − 0.26)**	**− 0.29 (− 0.47, − 0.12)**	**− 0.26 (− 0.45, − 0.07)**	**− 0.48 (− 0.85, − 0.12)**	**− 0.45 (− 0.53, − 0.37)**
Intercept	− 1.01 (− 1.37, − 0.66)	− 0.37 (− 0.74, − 0.01)	0.08 (− 0.39, 0.55)	1.04 (0.82, 1.28)	− 0.65 (− 1.00, − 0.31)	− 0.04 (− 0.26, 0.18)

*CrI* credible interval; bold fonts show 'statistically significant' results within a Bayesian framework (no zero within the 95% CrI)

Widely Applicable Information Criterion (WAIC) statistic was used to check the model fitness, the model which contains all the covariates was the best-fitting model for BCG immunization coverage in all EDHS surveys (see Additional file 1: Table S3).

## Discussion

This study found that BCG vaccination coverages were substantially varied at national, subnational, and local levels in Ethiopia. The spatial distribution of BCG vaccination coverage was also temporally varied. The overall BCG vaccination coverage in Ethiopia varies from53.5% in 2000, to 79.6% in 2016 and 79.0% in 2019, which indicates a 33% increase in the past two decades. The increment in the BCG vaccination coverage is partially due to the expansion and implementation of the health extension program p in Ethiopia. While the health extension program has several packages that promote disease prevention and control through household and community empowerment, immunization services including BCG vaccination were the core of the program [28]. Despite this effort, a slight decrement was observed between 2016 and 2019. These could be due to the internal instabilities and displacements of populations in some parts of the country, which might affect immunization services. The BCG vaccination coverage in our study is low as compared to the 2021 WHO report for Southeast Asia countries (87%) [29]. The low vaccination coverage in Ethiopia might be due to the difference in the socio-economic developments and healthcare systems for immunization programs. The recent COVID-19 pandemic might also affect immunization coverage in Ethiopia [30, 31].

Moreover, low predicted BCG vaccination coverage was observed in Afar, Benishangul, Gambela, and Somali regions [32]. Spatial clustering of TB was reported in these parts of the country in previous studies [33, 34]. This might indicate that in the area with low BCG vaccination there might be a high rate of TB incidence, an important finding to strengthen the BCG vaccination program in this part of the county. The possible reason for low BCG vaccination in Afar, Benishangul, Gambela, and Somali regions could be due to low healthcare access in these regions [35, 36]. Implementing a vaccination program and providing health education in these regions is also difficult as the people living in these areas are nomadic and seasonally move from place to place [37]. Therefore, special vaccination programs (such as mobile health services and immunization comping) that are well fitted with the lifestyle of the people are needed in this part of the country to increase the BCG vaccination coverage.

Our study also indicated that population density was positively associated with BCG vaccination coverage. Areas with high population density are mostly urban and health care access including immunization services is relatively good in urban areas [38]. Moreover, in areas with high population density mothers would have a better awareness of the benefits of childhood immunization services [39].

Seasonal variation is postulated as one of the factors related to immunization coverage. Healthcare-seeking behavior of the people would be low, particularly during the rainy and harvest season which may also affect the vaccination programs [40]. Routine healthcare services including immunization services would be low during the rainy seasons and cloudy environments, because of the difficulties in transportation as a result of flooding and muddy roads.

In line with previous studies conducted in Africa and other low and middle-income countries [41–43], distance to health facilities was negatively associated with BCG vaccination coverage in our study. This finding might indicate that poor infrastructure and low transportation services to access health facilities might be a bottleneck to improving vaccination coverage. [44–47]. In Ethiopia, approximately half of the population lives more than 10 km far from the nearest health facilities and has no access to immunization programs [48]. This inaccessibility of healthcare facilities would be a major barrier to immunization services improvements.

BCG vaccination coverage is also negatively associated with travel time to the nearest city, which is supported by studies conducted in rural Ethiopia [49], Madagascar [40], and Sudan [50]. As the travel time to the nearest cities increases, the accommodation and transportation expenses also increase, which would reduce the vaccination uptake of children.

This study has paramount implications for policymakers and health program designers to come up with evidence-based program enhancement to improve BCG vaccination coverage. However, the study had some important limitations; first, since, the study was based on secondary data, we could not include some important covariates such as literacy rate, and ANC coverage which might have an impact on the prediction of the BCG vaccine coverage. The other limitation of this study is the different data collection periods for the dependent and independent variables. Moreover, the BCG vaccine efficacy was not analyzed in this study due to the nature of the data and as it was beyond the scope of this study.

## Conclusion

The national BCG vaccination coverage was increased over time and varies substantially at the sub-national and local levels in Ethiopia. Low BCG vaccination coverage was observed in Southern, Southeastern, Northeastern, and Eastern parts of Ethiopia. Access to health facilities and travel time to the nearest cities were negatively associated with BCG vaccination coverage. Maintaining a high level of vaccination coverage across geographical areas is important to prevent TB in Ethiopia.

## Supplementary Information

**Additional file 1: Figure S1.** Uncertainty maps of BCG vaccination coverage among children under the age of five years in Ethiopia: A) 2000-2019, B) 2000, C) 2005, D) 2011, E) 2016 and F) 2019. **Table S1.** Covariate correlation result of variables included in this study. **Table S2.** Odds ratio with 95% Confidence Intervals (CI) of covariates included in a Bayesian spatial model with Binomial response for the BCG vaccination coverage in Ethiopia. **Table S3.** Watanabe-Akaike information criterion (WAIC) values corresponding to different model specifications. **Table S4.** Data sources and definitions of covariates.

## Data Availability

Relevant data is included within the manuscript and Additional file 1. If additional data is needed, it could be accessed by contacting the corresponding author. We have included the sources of all data in the Additional file 1 (see Additional file 1: Table S4).
