# Supplementary material for: Mapping BCG vaccination coverage in Ethiopia between 2000 and 2019
Source: BMC Infect Dis. 2022 Jun 23;22:569. doi: 10.1186/s12879-022-07547-4 (PMC9219134; doi:10.1186/s12879-022-07547-4)
Supplement: Supplementary file 1 — Additional file 1: Figure S1. Uncertainty maps of BCG vaccination coverage among children under the age of five years in Ethiopia: A) 2000-2019, B) 2000, C) 2005, D) 2011, E) 2016 and F) 2019. Table S1. Covariate correlation result of variables included in this study. Table S2. Odds ratio with 95% Confidence Intervals (CI) of covariates included in a Bayesian spatial model with Binomial response for the BCG vaccination coverage in Ethiopia. Table S3. Watanabe-Akaike information criterion (WAIC) values corresponding to different model specifications. Table S4. Data sources and definitions of covariates. [file 12879_2022_7547_MOESM1_ESM.docx]

**Supplementary information**

**
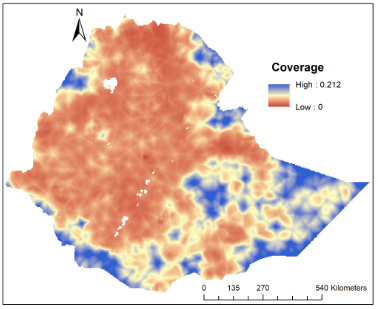
 A)
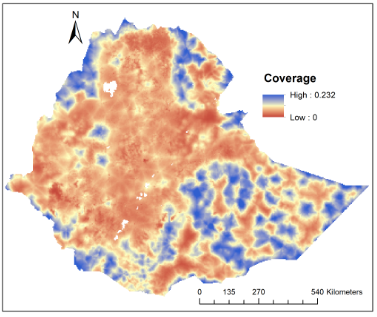
 B)**

**
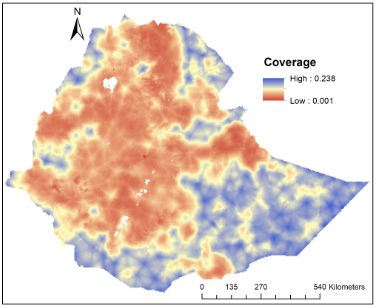
C)
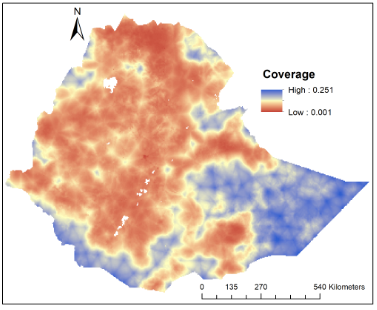
 D)**

**
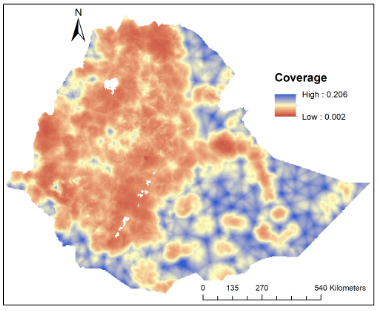
E)
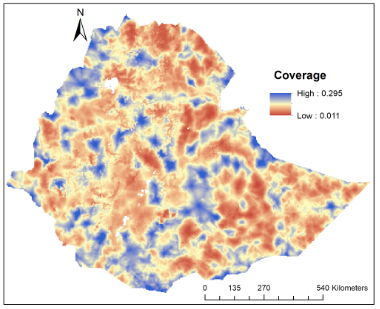
F)**

Figure S1: Uncertainty maps of BCG vaccination coverage among children under the age of five years in Ethiopia: *A) 2000-2019, B) 2000, C) 2005, D) 2011, E) 2016 and F) 2019*.

**Table S1: Covariate correlation result of variables included in this study**

| Covariates | Access to the health facility | Population density | Temperature | Precipitation | Access to city | Altitude | Distance to the water body |
| --- | --- | --- | --- | --- | --- | --- | --- |
| Access health facility | 1 | -0.19213 | 0.502995 | -0.52677 | 0.634061 | -0.52212 | -0.0721 |
| Population density | -0.19213 | 1 | -0.23901 | 0.178868 | -0.22035 | 0.244469 | 0.029721 |
| Temperature | 0.502995 | -0.23901 | 1 | -0.64523 | 0.450756 | -0.97829 | -0.19911 |
| Precipitation | -0.52677 | 0.178868 | -0.64523 | 1 | -0.39522 | 0.645797 | 0.168528 |
| Access city | 0.634061 | -0.22035 | 0.450756 | -0.39522 | 1 | -0.45208 | -0.04044 |
| Altitude | -0.52212 | 0.244469 | -0.97829 | 0.645797 | -0.45208 | 1 | 0.20824 |
| Distance to water body | -0.0721 | 0.029721 | -0.19911 | 0.168528 | -0.04044 | 0.20824 | 1 |

**Table S2:** Odds ratio with 95% Confidence Intervals (CI) of covariates included in a Bayesian spatial model with Binomial response for the BCG vaccination coverage in Ethiopia.

| Covariates | **Full immunization coverage**  Odds ratio with (95% CI) | | | | | |
| --- | --- | --- | --- | --- | --- | --- |
|  | **2000** | **2005** | **2011** | **2016** | **2019** | **2000-2019** |
| Temperature | 1.88(1.12, 3.16) | 1.43(0.87, 2.36) | 1.20(0.69, 2.10) | 1.31(0.74, 2.1) | 9.30(3.32, 26.31) | 1.19(0.92, 1.51) |
| Precipitation | 1.11(0.89, 1.39) | 0.83(0.64, 1.06) | 1.13(0.87, 1.48) | 1.17(0.93, 1.43) | 1.34(0.98, 1.80) | 0.95(0.84, 1.08) |
| Altitude | 1.63(0.95, 2.8) | 1.55(0.93, 2.59) | 1.34(0.76, 2.36) | 1.42(0.80, 2.48) | 13.40(4.81, 38.86) | 1.08(0.84, 1.39) |
| Travel time to the nearest city | 0.76(0.67, 1.16 | 0.79(0.68, 0.91) | 0.81(0.73, 0.91) | 0.88(0.76, 1.01) | 0.58(0.43, 0.77) | 0.89(0.84, 0.93) |
| Population density | 1.06(1.05, 1.07 | 1.05(1.03, 1.07) | 1.04(1.03, 1.06) | 1.03(1.02, 1.04) | 1.01(1.00, 1.03) | 1.05(1.04, 1.06) |
| Distance to water body | 0.96(0.91, 1.02) | 1.01(0.95, 1.06) | 0.95(0.90, 1.00) | 0.95(0.90, 1.02) | 0.92(0.81, 1.05) | 0.99(0.97, 1.02) |
| Distance to health facilities | 0.44(0.35, 1.79 | 0.63(0.51, 0.89) | 0.75(0.63, 0.89) | 0.77(0.64, 0.93) | 0.62(0.43, 0.89) | 0.64(0.59, 0.69) |
| Intercept | 0.36(0.25, 1.94 | 0.69(0.48, 0.99) | 1.08(0.68, 1.73) | 2.83(2.27, 3.60) | 0.52(0.37, 0.73) | 0.96(0.77, 1.20) |

**Table S3:** Watanabe-Akaike information criterion (WAIC) values corresponding to different model specifications.

| Model specifications | WAIC | | | | |  |
| --- | --- | --- | --- | --- | --- | --- |
|  | **2000** | **2005** | **2011** | **2016** | **2019** | **2000-2019** |
| Temperature | 3669.19 | 2979.48 | 3108.33 | 2313.55 | 1176.95 | 17126.74 |
| Temperature + Precipitation | 3641.51 | 2980.87 | 3106.98 | 2315.87 | 1179.09 | 17130.51 |
| Temperature + Precipitation + Altitude | 3639.28 | 2984.71 | 3112.54 | 2319.93 | 1164.74 | 17135.86 |
| Temperature + Precipitation + Altitude + Travel time | 3510.49 | 2949.94 | 3067.09 | 2309.04 | 1143.80 | 16946.82 |
| Temperature + Precipitation + Altitude + Travel time + Population density | 3233.86 | 2877.53 | 3019.31 | 2295.94 | 1153.89 | 16392.45 |
| Temperature + Precipitation + Altitude + Travel time + Population density + Distance to water body | 3235.70 | 2881.69 | 3016.46 | 2293.92 | 1155.50 | 16398.34 |
| Temperature + Precipitation + Altitude + Travel time + Population density + Distance to water body + Distance to health facilities | 3185.07 | 2863.79 | 3009.45 | 2292.63 | 1151.86 | 16278.29 |

**Table S4:** Data sources and definitions of covariates

| **Covariates** | **Data sources** | **Definitions** |
| --- | --- | --- |
| Population density | WorldPop | Number of people per square kilometre (grid) (1) |
| Travel times to cities | Malaria Atlas Project (MAP) | Travel time in minutes to the nearest city with a population of more than 50,000 (2) |
| Temperature | WorldClime | Annual mean environmental air temperature (°C) (3) |
| Precipitation | WorldClime | Annual mean rainfall (mm) (3) |
| Altitude | Shuttle Radar Topography Mission (SRTM) | Elevation of the earth land surface in km (4) |
| Distance to water body | Global Lakes and Wetlands Database (GLWD) | Distance to permanent and semi-permanent water based on presence of lakes, wetlands, rivers and streams, and accounting for slope and precipitation (5) |
| Access to healthcare facilities | Malaria Atlas Project (MAP) | Walking travel times in minutes to the nearest hospital or clinic (6) |

**References**

1. Tatem AJ. WorldPop, open data for spatial demography. Scientific data. 2017;4(1):1-4.

2. Weiss DJ, Nelson A, Gibson H, Temperley W, Peedell S, Lieber A, et al. A global map of travel time to cities to assess inequalities in accessibility in 2015. Nature. 2018;553(7688):333-6.

3. Fick SE, Hijmans RJ. WorldClim 2: new 1‐km spatial resolution climate surfaces for global land areas. International journal of climatology. 2017;37(12):4302-15.

4. Van Zyl JJ. The Shuttle Radar Topography Mission (SRTM): a breakthrough in remote sensing of topography. Acta Astronautica. 2001;48(5-12):559-65.

5. Lehner B, Döll P. Global lakes and wetlands database glwd. GLWD Docu mentation. 2004.

6. Weiss D, Nelson A, Vargas-Ruiz C, Gligorić K, Bavadekar S, Gabrilovich E, et al. Global maps of travel time to healthcare facilities. Nature Medicine. 2020;26(12):1835-8.
